# Effects of contrast-enhanced ultrasound treatment on neoadjuvant chemotherapy in breast cancer

**DOI:** 10.7150/thno.64767

**Published:** 2021-09-21

**Authors:** Anne Rix, Marion Piepenbrock, Barbara Flege, Saskia von Stillfried, Patrick Koczera, Tatjana Opacic, Nina Simons, Peter Boor, Sven Thoröe-Boveleth, Roel Deckers, Jan-Niklas May, Twan Lammers, Georg Schmitz, Elmar Stickeler, Fabian Kiessling

**Affiliations:** 1 Institute for Experimental Molecular Imaging, Medical Faculty, RWTH Aachen University, Aachen, Germany.; 2 Chair for Medical Engineering, Department of Electrical Engineering and Information Technology, Ruhr University Bochum, Bochum, Germany.; 3 Department of Obstetrics and Gynecology, Medical Faculty, RWTH Aachen University, Aachen, Germany.; 4 Institute of Pathology, Medical Faculty, RWTH Aachen University, Aachen, Germany.; 5 Department of Intensive Care and Intermediate Care, Medical Faculty, University Hospital RWTH Aachen, Aachen, Germany.; 6 Institute for Occupational, Social and Environmental Medicine; Medical Faculty, RWTH Aachen University, Aachen, Germany.; 7 Division of Imaging and Oncology, University Medical Center Utrecht, Utrecht, The Netherlands.

**Keywords:** CEUS, chemotherapy, breast cancer, super-resolution ultrasound, sonopermeation

## Abstract

**Purpose:** Preclinical and clinical data indicate that contrast-enhanced ultrasound can enhance tumor perfusion and vessel permeability, thus, improving chemotherapy accumulation and therapeutic outcome. Therefore, we investigated the effects of high mechanical index (MI) contrast-enhanced Doppler ultrasound (CDUS) on tumor perfusion in breast cancer.

**Methods:** In this prospective study, breast cancer patients were randomly assigned to receive either 18 minutes of high MI CDUS during chemotherapy infusion (n = 6) or chemotherapy alone (n = 5). Tumor perfusion was measured before and after at least six chemotherapy cycles using motion-model ultrasound localization microscopy. Additionally, acute effects of CDUS on vessel perfusion and chemotherapy distribution were evaluated in mice bearing triple-negative breast cancer (TNBC).

**Results:** Morphological and functional vascular characteristics of breast cancer in patients were not significantly influenced by high MI CDUS. However, complete clinical tumor response after neoadjuvant chemotherapy was lower in high MI CDUS-treated (1/6) compared to untreated patients (4/5) and size reduction of high MI CDUS treated tumors tended to be delayed at early chemotherapy cycles. In mice with TNBC high MI CDUS decreased the perfused tumor vessel fraction (p < 0.01) without affecting carboplatin accumulation or distribution. Higher vascular immaturity and lower stromal stabilization may explain the stronger vascular response in murine than human tumors.

**Conclusion:** High MI CDUS had no detectable effect on breast cancer vascularization in patients. In mice, the same high MI CDUS setting did not affect chemotherapy accumulation although strong effects on the tumor vasculature were detected histologically. Thus, sonopermeabilization in human breast cancers might not be effective using high MI CDUS protocols and future applications may rather focus on low MI approaches triggering microbubble oscillations instead of destruction. Furthermore, our results show that there are profound differences in the response of mouse and human tumor vasculature to high MI CDUS, which need to be further explored and considered in clinical translation.

## Introduction

Sonoporation, or sonopermeation, describes the opening of cellular membranes or tissues by acoustic waves. Microbubbles can enhance sonoporation effects on cellular membranes caused by physical forces (e.g., microstreaming, jet formation, shock waves) from their oscillation or destruction in the acoustic field. Microbubble-assisted ultrasound techniques were successfully applied in several *in vitro* and *in vivo* experiments to permeabilize cellular membranes or temporarily open tight junctions between endothelial cells to enhance the delivery of therapeutic molecules into a target tissue 1-3. In this regard, a particular focus was set on opening the blood-brain barrier to enhance drug delivery to brain tumors, as the blood-brain barrier prevents most of the therapeutic molecules from entering the brain. Aryal and colleagues showed a 2-fold increase in the accumulation of liposomal doxorubicin in gliomas in rats after co-administration with microbubbles and application of focused ultrasound 4. Furthermore, Carpentier and colleagues showed a successful opening of the blood-brain barrier in a phase I clinical trial, in which glioblastoma patients were repeatedly treated with microbubbles and an implantable ultrasound device, followed by intravenous administration of carboplatin. No progression of the tumor area exposed to the ultrasound beam was detected by magnetic resonance imaging in 9 out of 11 patients with confirmed blood-brain barrier disruption. All four patients without confirmed blood-brain barrier disruption demonstrated tumor progression 5.

Apart from applications for the treatment of brain tumors, a combination of chemotherapeutic agents and microbubble-assisted ultrasound (sonochemotherapy) has been used in tumors outside the brain. In this regard, Yousefian and colleagues showed a tumor growth delay of murine breast cancer after a combination of bleomycin injection and microbubble-assisted ultrasound, compared to untreated, bleomycin alone or ultrasound alone treated tumors 6. Besides small therapeutic molecules like bleomycin, it could be demonstrated that the co-administration of microbubbles and doxorubicin loaded liposomes with a size of approximately 100 nm in combination with ultrasound could enhance the liposome accumulation in a mouse neuroblastoma tumor model 2 and improve the treatment response of murine colorectal cancer 7. In a first clinical phase I study, pancreatic ductal adenocarcinoma patients treated with microbubble-assisted ultrasound could receive a higher number of treatment cycles with gemcitabine compared to a historical cohort treated with gemcitabine alone. Five out of ten treated patients showed tumor size reduction and were evaluated for consolidation radiation therapy or further chemotherapy with FOLFIRINOX 8. It was hypothesized that the improved therapeutic efficacy might result from increased gemcitabine uptake and deeper penetration into the tumor tissue in combination with an enhanced drug sensitivity of the tumor cells. Furthermore, Theek and colleagues showed that sonopermeation of desmoplastic tumors in mice does not only increase the accumulation but also the penetration of liposomes 9.

These findings could be especially interesting for patients diagnosed with desmoplastic tumors (e.g., pancreatic or breast cancer) that have a poor prognosis. With more than two million cases in 2018, breast cancer is the second most common cancer worldwide 10. Ten to fifteen percent of these patients are diagnosed with triple-negative breast cancer (TNBC), defined by a lack of expression of the progesterone receptor, estrogen receptor, and human epidermal growth factor receptor 2. Due to the lack of these receptors, classical targeted therapies cannot be applied, and neoadjuvant chemotherapy is the main systemic treatment strategy. Furthermore, TNBC is associated with the worst prognosis of all breast cancer subtypes regarding disease-free and overall survival. Patients with TNBC who do achieve a pathological complete response (pCR) after neoadjuvant chemotherapy show better progression-free survival (75% lower risk of recurrence 11) as well as overall survival than patients with residual disease 12. Therefore, treatment of breast cancer, especially TNBC, could benefit from strategies or treatment modifications aiming to enhance therapy response by improved drug accumulation in the tumor.

One reason for the poor prognosis is the restriction of cytostatic drug delivery to the tumors. The distribution of drugs in tumor tissues does not only depend on the drugs' physicochemical characteristics but also on the composition of the tumors, e.g., density and assembly of tumor cells, the composition of the extracellular matrix, size of the interstitial space, interstitial fluid pressure, and vascularization 13. The prominent stromal barrier is one factor that limits the accumulation of chemotherapeutics in the tumor. In this context, it was shown that increased tumor perfusion correlated with improved response to chemotherapy in preclinical breast cancer models 14 and with progression-free and improved overall survival in glioblastoma patients 15. The tumor perfusion can be assessed by contrast-enhanced imaging, e.g., computed tomography, magnetic resonance imaging, or ultrasound.

In this regard, ultrasound is routinely used as an imaging technique for the breast and applied as a monitoring tool for patients undergoing neoadjuvant treatment of breast cancer. Several clinical studies show the feasibility of contrast-enhanced ultrasound (CEUS) for breast cancer diagnosis 16 and monitoring of treatment response 17, 18. In Europe, the use of microbubbles to improve the detectability of the vasculature in focal lesions of the liver and breast with contrast-enhanced Doppler ultrasound (CDUS) is approved by the European Medicines Agency (e. g., for SonoVue® under EMA EU/1/01/177/002).

Contrast-enhanced (Doppler) ultrasound takes advantage of the physical effects of microbubbles as it aims to generate microbubble oscillations 19 or even destruction 20. In the case of harmonic CEUS, nonlinear acoustic responses of the microbubbles provide specific microbubble signatures, ensuring an excellent detection sensitivity and specificity. Furthermore, in CDUS, microbubbles can be detected either by their backscatter properties or by their collapse in high mechanical index (MI) power Doppler as a pseudo-Doppler signal 21.

Similarly, but with a therapeutic intention, sonopermeation aims at microbubble oscillation- or destruction-induced opening of biological barriers, such as vascular walls. Most preclinical and clinical sonopermeation studies were performed at a relatively low frequency of 1 - 2 MHz or below 2, 4-7. However, an increased accumulation of therapeutic molecules was also observed at high frequencies of 16 MHz 9. Consequently, already diagnostic harmonic CEUS or CDUS may probably have a sonopermeation effect on tumors, enhancing chemotherapy accumulation and therapeutic efficacy.

Taking this notion into account, we performed a prospective clinical study to investigate whether the repeated application of contrast-enhanced Doppler ultrasound with destruction of microbubbles during chemotherapy infusion influences tumor perfusion to improve the response of breast cancer to neoadjuvant chemotherapy. In this context, super-resolution ultrasound imaging (motion-model ultrasound localization microscopy (mULM)) 22 was applied to sensitively assess functional changes in the tumor vascularization during chemotherapy. We found no effects on perfusion of breast cancer in patients during our investigation, and even observed a tendency of an initially delayed tumor size reduction during neoadjuvant chemotherapy. Therefore, the recruitment of patients was suspended, and further preclinical experiments on a murine TNBC model were performed with identical ultrasound settings to unravel the effects of high MI CDUS mechanistically. The direct comparison of preclinical and clinical examinations enabled us to identify different factors that might impede a successful clinical translation of microbubble assisted ultrasound treatment and should be considered in the planning of future experiments.

## Materials & Methods

### Patient recruitment

The study was approved by the RWTH Aachen University Hospital's ethics board and registered in clinicaltrials.gov (NCT03385200). All patients gave their written consent to participate. A detailed description of the clinical trial can be found in the supplementary information (supplementary table S1 & supplementary figure S1). Eleven female patients (ages 34-56 years) with histologically confirmed primary breast cancer (TNBC, Her2neu positive, or hormone receptor-positive tumors, Supplementary Table S2) who received neoadjuvant chemotherapy were included. TNBC patients received four cycles of epirubicin (90 mg/m^2^) + cyclophosphamide (600 mg/m^2^, Endoxan, Baxter Oncology, Germany) every 2 - 3 weeks, followed by 12 weekly cycles of a combination of paclitaxel (80 mg/m^2^, Ribotax®, Ribosepharm, Germany) with carboplatin (1.5 mg/ml/min over 1 h, CarboCell®, STADA, Germany). Treatment schemes of patients with Her2neu positive and hormone receptor positive tumors can be found in the methods part of the supplementary information. Before chemotherapy infusion, the patients' health condition and blood count were analyzed according to our oncological day clinics' local standards. They received a defined, protocol-specific premedication consisting of dexamethasone, ranitidine, and dimetindene.

Before the first chemotherapy cycle, patients were randomly assigned to receive either only standard chemotherapy treatment (CTx; n = 5) or additionally high MI contrast-enhanced Doppler ultrasound (CTx + high MI CDUS; n = 6).

### Tumor perfusion assessment with contrast-enhanced ultrasound

Before each chemotherapy infusion, tumor sizes were measured using the Toshiba Aplio 500 ultrasound scanner (14L5 1005BT transducer) in B-mode. Tumor volumes were calculated using the formula W x H x D x π / 6. Next, tumor perfusion was assessed in non-destructive contrast specific mode (5 MHz, MI 0.07, thermal index (TI) < 0.4, framerate 15/sec, persistence 0, gain and dynamic range were kept constant for all measurements) for 180 seconds by measuring the inflow of 0.5 ml (1-5 x 10^8^ microbubbles/ml 23) of the clinically used contrast-agent SonoVue^®^ (Bracco, Milan, Italy). The contrast agent was slowly injected intravenously in a permanent venous port system, which is always placed into the subclavian vein. Only one central slice of the tumor was imaged at a low MI to reduce the effects of the perfusion measurement on the tumor to a minimum. Perfusion measurements were repeated directly after each chemotherapy cycle.

### High mechanical index contrast-enhanced Doppler ultrasound (high MI CDUS)

**Figure 1:**
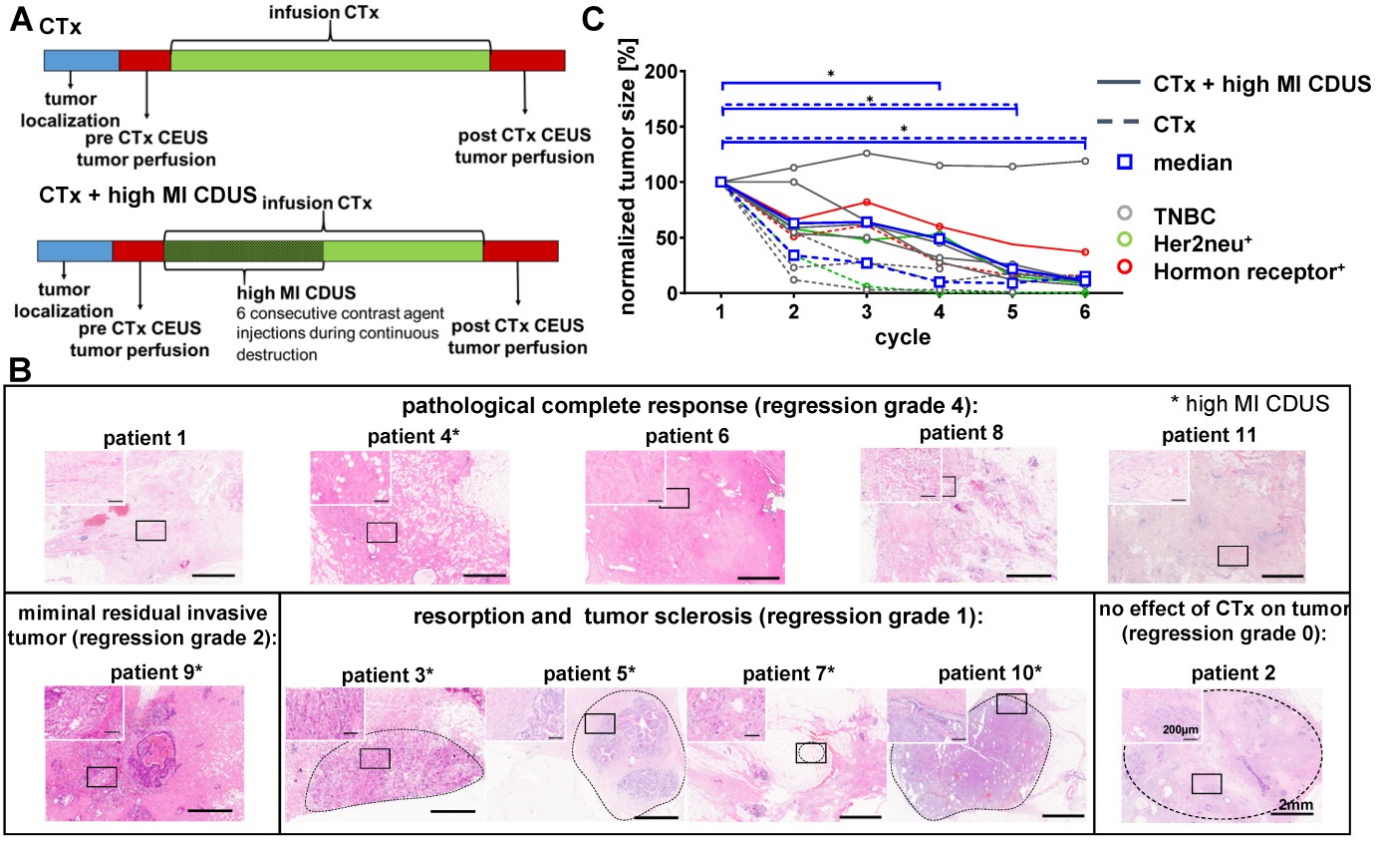
** Patient responses to neoadjuvant chemotherapy with and without contrast-enhanced ultrasound treatment. A:** Experimental design: tumor size was measured before chemotherapy (CTx) infusion. Before and after chemotherapy, the assessment of tumor perfusion was performed by contrast-enhanced ultrasound (CEUS). In patients receiving CTx + high MI CDUS, 18 min of high MI CDUS was applied to the tumor during CTx infusion. **B:** Representative H&E staining of the tumor region after surgical resection. Patients marked with an asterisk received CTx + high MI CDUS. C: CTx-treated tumors (n = 5) showed a significant tumor size reduction from the 4^th^ cycle of chemotherapy onwards. CTx + high MI CDUS treated tumors (n = 6) responded more slowly to chemotherapy, and tumor size reduction failed significance during the first four cycles due to one non-responding tumor.

In patients receiving high MI CDUS, destructive contrast-enhanced ultrasound at a high MI (MI 0.8) was applied on the whole tumor during the first 18 minutes of chemotherapy infusion in Doppler mode (80% power) using a Philips iU22 ultrasound system (Philips, Eindhoven, Netherlands) equipped with an L17-5 transducer working at 7 MHz (pulse lengths approximately 0.008 ms, 7% duty cycle). During this time, six consecutive slow intravenous injections of 0.5 ml SonoVue^®^ were performed every 3 min. To enable a homogenous treatment of the whole tumor, the transducer was continuously moved over the entire tumor volume. Before and after each injection, Doppler images of the tumor were recorded. The percentage of Doppler positive area in relation to the whole tumor area was determined to estimate tumor vascularization using the Imalytics Preclinical Software 24. A graphical illustration of the treatment schedule can be found in Figure 1A.

### Time-intensity curve analysis and motion-model ultrasound localization microscopy (mULM)

Post-processing of the acquired ultrasound images was performed as described previously 22. The in-plane tissue motion between one reference frame and each frame of the B-mode sequence was computed with an affine motion correction algorithm. The frame with the highest normalized cross-correlation (NCC) to each frame of the sequence was selected as a reference frame. Then, the non-linear contrast mode was corrected for the estimated tissue motion. Afterward, the NCC between the reference frame and each motion corrected B-mode image was computed. For frames with a successful in-plane motion correction, the NCC to the reference frame increases, while frames with out-of-plane motion still show a low NCC and were identified and eliminated before further processing. To determine the peak enhancement (PE), motion-corrected data from perfusion measurements were converted into time-intensity curves. PE was calculated as the difference from the baseline values to the highest signal enhancement.

The motion-corrected contrast mode images were used to localize single microbubbles on a highly resolved grid via gauss-detection 25. The contrast mode images were interpolated with a spline interpolation to a 10 μm-grid and convolved with a Gaussian kernel that matches the size of the ultrasound system's point-spread-function (σ = 335 μm) 26. After thresholding, maxima were identified to get the highly resolved microbubble positions. For preclinical data, a median filter was applied to the motion corrected B-mode images to separate moving microbubbles from the static tissue 26, 27, and the Gauss detection was applied.

The vasculature was reconstructed by tracking microbubbles in consecutive frames. The tracking was performed by the Markov chain Monte Carlo data association algorithm (MCMCDA) because of its robustness in case of complex circumstances, like a low frame rate or crossing microbubble tracks due to a low elevational resolution 25, 26. The MCMCDA assigns microbubbles to tracks and aims to find an association of the detected positions with tracks that have the maximum a posteriori probability under the observations. The reconstructed vasculature was then visualized dependent on the number of detected tracks per pixel (count map), the flow directions, and the flow velocities. Furthermore, the relative blood volume (rBV), mean velocity, and the respective standard deviation were evaluated for each dataset. The rBV was computed from the count maps with the estimator from 27 to normalize to the acquisition length.

### Preclinical evaluation of carboplatin accumulation in murine triple-negative breast cancer

The German State Office for Nature, Environment and Consumer Protection (LANUV) North Rhine-Westphalia approved all animal experiments. A total of 15 female Balb/cAnNRj mice (age 10-12 weeks; Janvier Labs, Saint Berthevin, France) were housed in groups of 5 animals on spruce granulate bedding (Lignocel, JRS, Germany) under specific pathogen-free conditions with a 12 h light/dark cycle in a temperature (20-24 °C) and humidity (45-65%) controlled environment according to the guidelines of the “Federation for Laboratory Science Associations” (FELASA). Standard pellets (Sniff GmbH, Soest, Germany) and acidified water were offered ad libitum.

After an acclimatization period of 7 days, 4 x 10^4^ murine syngeneic TNBC cells (4T1, ATCC, Manassas, USA, RRID:CVCL_0125) in 50 µl RPMI 1640 (ThermoFisher, Waltham, USA) medium were orthotopically injected into the right inguinal fat pad. Tumor size was assessed daily via caliper. When tumors reached 6 - 8 mm in diameter, mice were randomly allocated to three experimental groups (n = 5 per group). Animals of the first group (CTx) received an intravenous infusion of 50 mg/kg bodyweight carboplatin (CarboCell®, STADA, Germany) in 0.9% sterile saline with an infusion rate of 0.01 ml/min for 10 min in a lateral tail vein. The second group (CTx + MB) was treated with five consecutive intravenous injections of 1 x 10^5^ microbubbles SonoVue^®^ in 25 µl 0.9% sterile saline every 2 min, additionally to carboplatin infusion. Animals of the third group (CTx + high MI CDUS) received the carboplatin infusion and five consecutive intravenous injections of 1 × 10^5^ microbubbles SonoVue^®^ in 25 µl 0.9% sterile saline every 2 min while high MI CDUS was performed using the Philips iU22 ultrasound system (Philips, Eindhoven, Netherlands, L17-5 transducer, 7 MHz, MI 0.8). The duration of high MI CDUS was reduced to 10 min to ensure equivalent total ultrasound energy deposition in the smaller murine tumors compared to human tumors. The 2 min interval between microbubble injections was chosen as a compromise between a faster clearance of microbubbles due to the higher heartrate in mice and an even distribution of the injections over the 10 min treatment time. The distance of the transducer surface to the mouse tumors was adjusted to 1.5 cm (representing the mean imaging depth in humans) using ultrasound gel (Arne Maass, Borken, Germany). Additionally, the inflow of 1 × 10^5^ microbubbles SonoVue^®^ in 25 µl 0.9% NaCl was imaged using the Vevo3100 (VisualSonics, Toronto, Canada) small animal imaging system equipped with the MX250 transducer in contrast mode (18 MHz, MI 0.07, framerate 10/sec) to assess the tumor perfusion before and after high MI CDUS.

After completing the carboplatin infusion, 60 µl rhodamine-labeled* Ricinus Communis*-agglutinin I (5 mg/ml Vector laboratories, Burlingame, USA) were intravenously injected to stain perfused vessels in the tumor followed by cervical dislocation to euthanize the animals.

Murine tumors were collected and bisected. One half was embedded in Tissue Tek® O.C.T. Compound (Sakura, Alphen aan den Rijn, Netherlands), the other half was snap-frozen for ICP-MS analysis, and both were stored at -80 °C.

For comparison of tumors between mouse and human TNBC, histological specimens of 10 untreated 4T1 tumors grown in the same mouse strain were analyzed. Half was embedded in Tissue Tek O.C.T. compound; the other half was embedded in paraffin. Paraffin-embedded murine tumor samples were cut in 5 µm thick slices and stained for connective tissue. Immunofluorescence staining of tumor vasculature was performed on frozen tissue samples. A detailed description of ICP-MS analyses and histological staining can be found in the supplementary information.

### Histological and immunofluorescence staining

Paraffin-embedded human tumor samples were cut in 3 µm thick slices. Staining for tissue morphology (hematoxylin & eosin (H&E)) and connective tissue (trichrome staining) were performed. Furthermore, immunofluorescence staining of tumor vasculature was performed. A detailed description of staining procedures can be found in the supplementary information.

### Statistical analyses

All data are presented as the median and interquartile range (interquartile 1 - interquartile 3). Statistical analyses were performed using Graph Pad Prism 9.0 (Graph Pad Software, San Diego, USA). Differences in tumor size and ultrasound parameters between CTx and CTx + high MI CDUS treated patients at the single time points were tested for statistical significance using the Mann-Whitney test. The number of animals per group was calculated using a power calculation 28, assuming that tumor perfusion differences can be detected with 80% power. Preclinical data were analyzed by Kruskal-Wallis with Dunn's post-test or by Wilcoxon signed-rank test for paired data. P < 0.05 was considered to indicate significant differences.

## Results

### Patient responses to chemotherapy

Tumor sizes were comparable between CTx and CTx + high MI CDUS treated patients (Supplementary Figure S2) and decreased in all patients during chemotherapy, as assessed by B-mode ultrasound imaging before each chemotherapy cycle. Three patients treated with CTx and two patients treated with CTx + high MI CDUS showed a clinical complete response, and tumors were not detectable by ultrasound after seven chemotherapy cycles anymore. In two patients of the CTx and four of the CTx + high MI CDUS group, tumor tissue could be visualized by ultrasound until the end of neoadjuvant treatment. Histologically, after surgical resection, five patients had a pathological complete response (pCR) (4 CTx, 1 CTx + high MI CDUS, regression grade 4), a minimal residual invasive tumor was diagnosed in one patient (CTx + high MI CDUS), four patients showed resorption and tumor sclerosis (all CTx + high MI CDUS, regression grade 1), and one patient no effect of chemotherapy (CTx, regression grade 0) (Figure 1B, Supplementary Table S3). A comparison of the initial tumor size and vascularization with regression grade did not show any connection of these parameters (Supplementary Figure S3).

Despite the overall tumor response, a delayed tumor size reduction of high MI CDUS treated tumors during the first chemotherapy cycles was detected. In detail, a significant decrease in tumor size (p < 0.05) could be observed from the fourth cycle of chemotherapy onwards in CTx treated patients, whereas tumors of CTx + high MI CDUS treated patients showed significant changes from the fifth cycle of chemotherapy onwards due to one non-responding tumor (Figure 1C).

This tendency towards a delayed response of tumors to chemotherapy after high MI CDUS resulted in the decision to pause the clinical study.

### Response of tumor vascularization to high MI CDUS in patients

Almost fifty percent of the tumors were not detectable by ultrasound at cycle seven of chemotherapy anymore. Therefore, only data from the first six cycles were used to compare CTx, and CTx + high MI CDUS treated patients.

**Figure 2:**
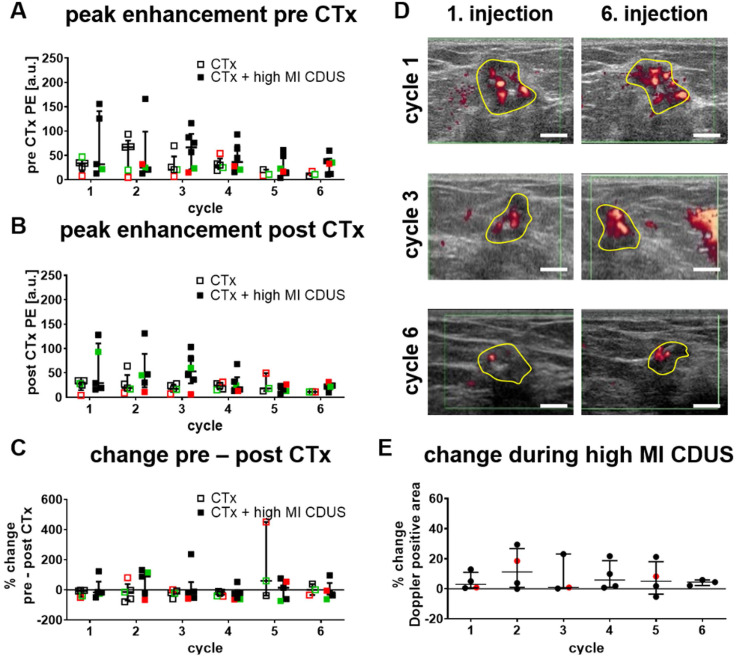
** Response of tumor vascularization to high MI CDUS in patients. A & B:** No longitudinal changes in the tumor vascularization, assessed by peak enhancement (PE) between tumors receiving chemotherapy alone (CTx; n = 5) or in combination with high MI CDUS (CTx + high MI CDUS; n = 6), are detected. (black = TNBC, green = Her2neu positive, red = hormone receptor positive) **C:** The percentage change between PE before and directly after chemotherapy infusion does not indicate an effect of high MI CDUS. **D:** Representative Doppler images after the 1^st^ and 6^th^ microbubble injection during high MI CDUS treatment do not indicate substantial changes in tumor perfusion (tumor area outlined in yellow; scale bar 0.5 cm). **E:** There is no noticeable difference in the Doppler positive area between the 1^st^ and 6^th^ microbubble injection, indicating that tumor perfusion does not substantially change during high MI CDUS treatment (all values are presented as the median and interquartile range).

We first searched for longitudinal effects of high MI CDUS on tumor vascularization by comparing the relative PE of CTx and CTx + high MI CDUS treated patients before (Figure 2A) and after (Figure 2B) each chemotherapy cycle. There were no significant differences between the groups in both cases, and mean PE values remained almost stable until they tended to decrease after the 4^th^ chemotherapy cycle. Furthermore, the PE was comparable between the different tumor types.

Next, changes in tumor vascularization before and directly after high MI CDUS were investigated by comparing the PE values after each chemotherapy infusion with the respective value acquired before chemotherapy. As indicated in Figure 2C, PE values did not significantly change after chemotherapy administration, independent of the application of high MI CDUS.

Furthermore, the change in Doppler signals during high MI CDUS was assessed in CTx + high MI CDUS treated patients to investigate the occurrence of immediate effects on the tumor vascularization. The analysis of the Doppler positive tumor area did not reveal a clear trend towards changed perfusion (Figure 2D & E).

### Motion model ultrasound localization microscopy (mULM)

**Figure 3:**
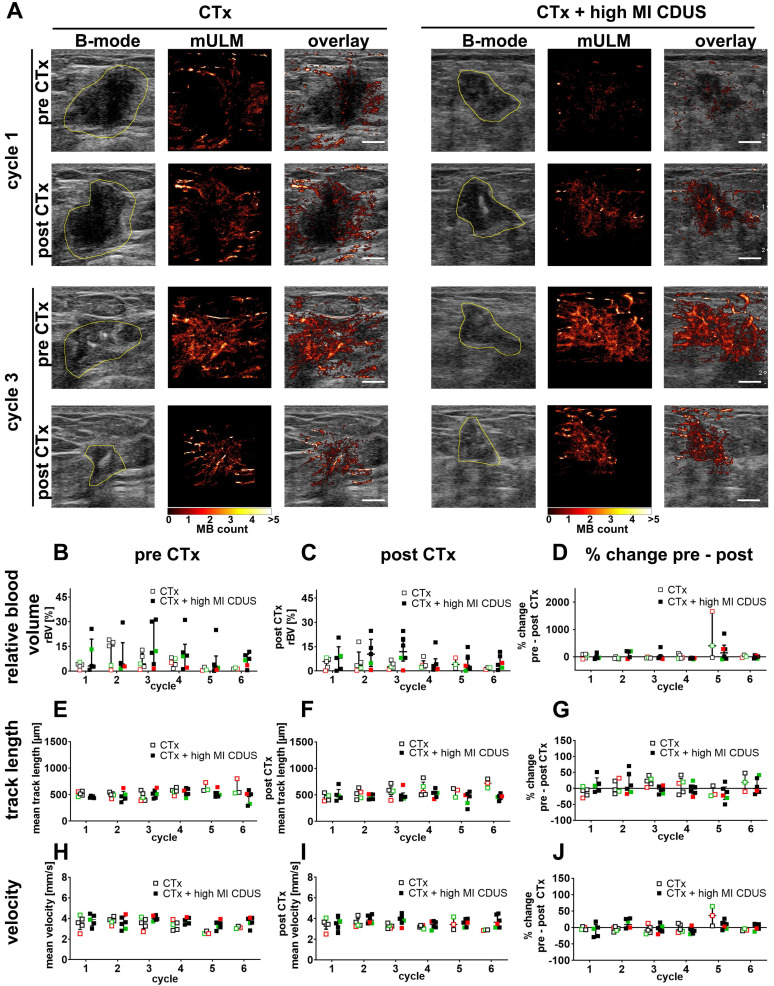
** Motion model ultrasound localization microscopy (mULM) in patients.** mULM was applied to assess discrete morphological and functional changes in the tumor vasculature. **A:** Representative B-mode images and the respective microbubble tracks assessed by mULM of CTx and CTx + high MI CDUS treated tumors before and after chemotherapy infusion at cycles 1 and 3 of chemotherapy show an increased rBV at cycle 3 in both groups. Tumor margins are outlined in yellow (scale bar: 0.5 cm). **B-J:** No significant effects of high MI CDUS are detected neither when comparing the high MI CDUS group (n = 6) with the CTx group (n = 5) nor the percentage change from pre and post CTx values of both groups concerning rBV (**B, C, D**), track length (**E, F, G**), and microbubble velocity (**H, I, J**). All data are presented as the median and interquartile range (black = TNBC, green = Her2neu positive, red = hormone receptor positive).

To identify subtle differences in vascularization between CTx and CTx + high MI CDUS treated tumors, mULM was applied. We first analyzed the effects on rBV. Compared to PE, the rBV is a more robust parameter in detecting tumor vascularization as it is not influenced by the injection speed of microbubbles. In line with PE results, there were no significant alterations in rBV from before to after chemotherapy infusion, no significant changes over the treatment period, and comparable values were obtained for CTx and CTx + high MI CDUS treated tumors (Figure 3A-D).

Furthermore, we did not observe any significant differences in mean track length, where a reduction would indicate a vascular breakdown (Figure 3A, E-G). Finally, the velocity of microbubbles was assessed as a measure of tumor perfusion (Figure 3A, H-J). Again, values were comparable between tumor types, groups and time points, indicating no measurable differences in vascular morphology and function.

### Preclinical evaluation of high MI CDUS effects on tumors

**Figure 4:**
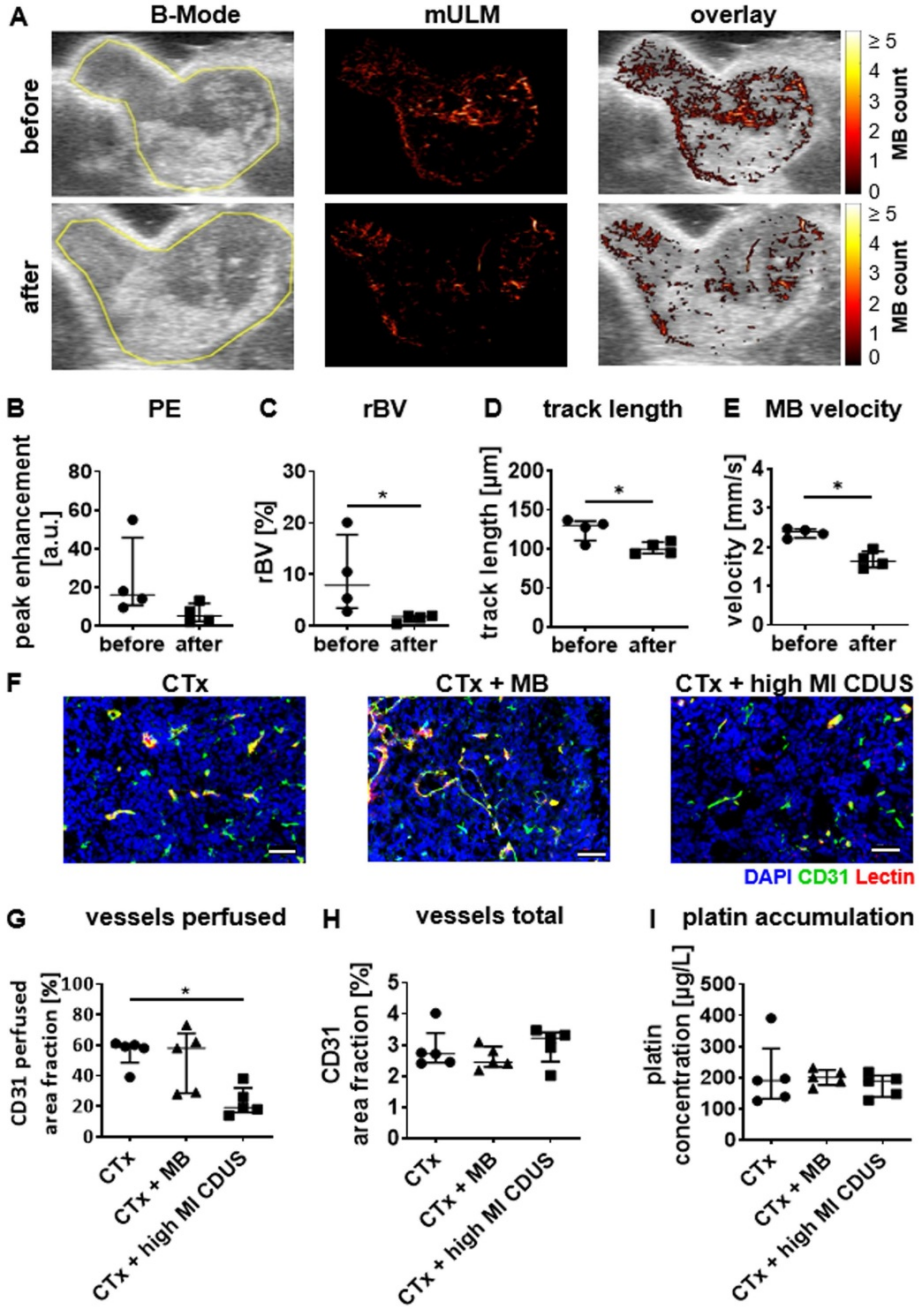
** High MI CDUS does not affect carboplatin accumulation in murine 4T1 tumors. A:** Representative images of tumor rBV assessed by mULM before and after high MI CDUS indicating vascular breakdown (tumor is outlined in yellow). In mice, the application of 10 min high MI CDUS during carboplatin infusion significantly decreases **B:** peak enhancement (PE), **C:** relative blood volume (rBV), **D:** track length, and **E:** microbubble velocity directly after CTx + high MI CDUS. **F:** Representative immunofluorescence micrographs of the tumor vascularization. Scale bar: 50 µm. **G:** Vascular breakdown was confirmed by immunofluorescent analysis, where significantly less perfused vessels (assessed as the percentage of CD31 and lectin douple-positive vessels with all CD31 positive vessels) are detected. **H:** The total vessel density, measured as CD31-positive are fraction, is comparable in all treatment groups. **I:** Although CTx + high MI CDUS treated tumors show less perfused vessels, a comparable accumulation of carboplatin was found by ICP-MS in all treatment groups (all values are presented as median and interquartile range. n = 5 animals per group * = p < 0.05).

In mice with 4T1 tumors, conventional ultrasound analysis showed a slight reduction in PE after CTx + high MI CDUS treatment of 4T1 tumors (Figure 4A & B). In line with this, mULM depicted a significant decrease in rBV (before: 7.9 (3.4-17.7)%; after: 1.8 (0.7-1.9)%; p = 0.0286 Figure 4A & C), track length (before: 130 (111-136) µm; after: 100 (94-109) µm: p = 0.0488; Figure 4A & D) and microbubble velocity (before: 2.39 (2.24-2.46) mm/s; after: 1.64 (1.48-1.89) mm/s; p = 0.0286; Figure 4A & E) when comparing measurements before and after carboplatin infusion in tumors receiving CTx + high MI CDUS. These findings were confirmed by histological analysis revealing a significantly lower percentage of perfused vessels after CTx + high MI CDUS compared to tumors that only received carboplatin infusion (CTx: 50 (48.5-60.5)%; CTx + MB: 58 (28.5-67.5)%; CTx + high MI CDUS: 19 (16.0-32.0)%; p = 0.0079; Figure 4F & G). As the total number of CD31-positive vessels was comparable in all treatment groups (Figure 4F & H), this points towards an acute effect on vessel functionality. Interestingly, despite the decreased tumor perfusion, the amount and distribution of carboplatin in the tumors assessed by ICP-MS was comparable in all groups (Figure 4I & Supplementary Figure S4).

**Figure 5:**
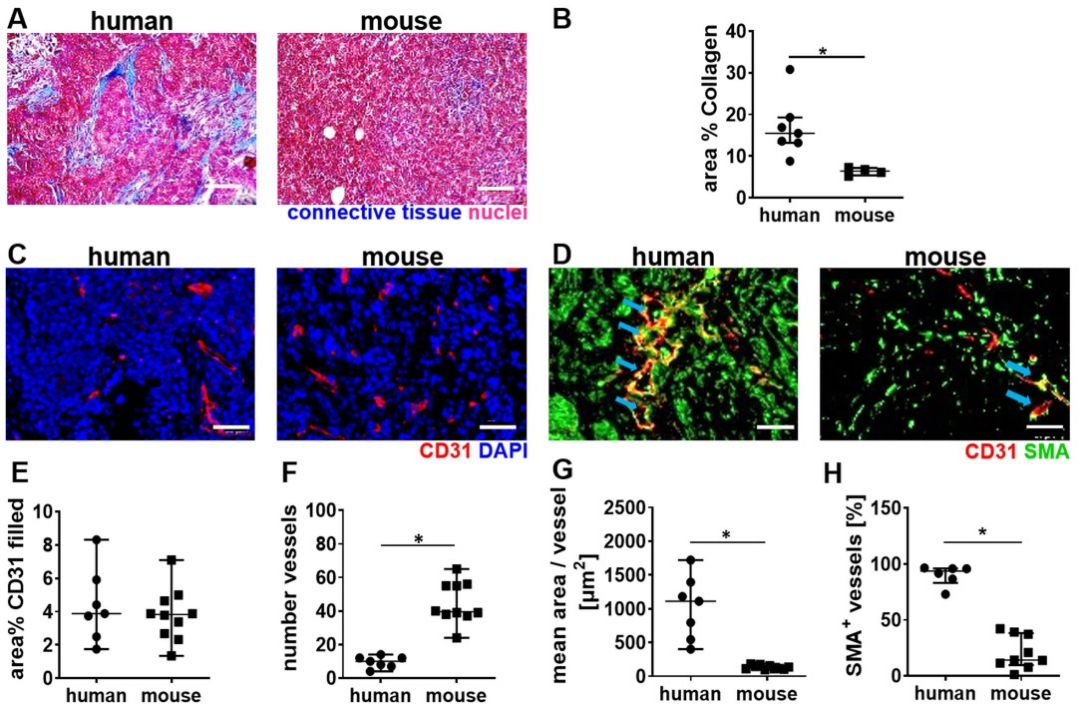
** Differences in tumor vascularization in human TNBC and murine 4T1 tumors. A:** Trichrome staining of murine (n = 5) and human TNBC (n = 7). **B:** The quantification of trichrome staining indicates more connective tissue in human TNBC than murine 4T1 tumors. **C:** Representative immunofluorescence micrographs of the vasculature in human TNBC compared to murine 4T1 tumors (scale bar 50 µm). **D:** Representative immunofluorescence micrographs show the vessel maturity in human TNBC, compared to murine 4T1 tumors (scale bar 50 µm) blue arrows indicate SMA^+^ vessels. **E:** The filled area fraction of CD31-positive vessels is comparable between human (n = 7) and murine (n = 10) TNBC. **F:** Murine 4T1 tumors contain significantly more CD31 positive vessels than human TNBC. **G:** Calculating the mean area per CD31-positive vessel reveals that human TNBC contain significantly larger vessels than murine 4T1 tumors. H: Human TNBC contain significantly more mature (SMA positive) vessels than murine 4T1 tumors. All values are presented as median and interquartile range. * = p < 0.05.

Next, a histological comparison of human and mouse tumor tissue was performed to understand the stronger vascular response of the murine TNBC to high MI CDUS. We found that human TNBC lesions contain more connective tissue, which is often localized around the tumor microvessels. In contrast, murine 4T1 tumors contain only a very low amount of connective tissue, and microvessels are in closer contact with tumor cells (Figure 5A & B). The analysis of tumor microvessels revealed a comparable CD31 filled area fraction in both species (human: 3.8 (2.6-5.5)%; mouse: 3.8 (2.6-4.7)%) (Figure 5C & E). However, murine tumors contained significantly more (human: 9 (7-12); mouse: 40 (38-55); p = 0.0004; Figure 5C & F) but smaller microvessels compared to human tumors (human: 954 (558-1340) µm^2^; mouse: 127 (110-160) µm^2^; p < 0.0001; Figure 5C & G). Furthermore, the vessel maturity was significantly higher in human TNBC compared to murine TNBC (human: 93.7 (83.1-95.9)%; mouse: 14.1 (9.6-38.2)%; p = 0.0004) (Figure 5D & H).

## Discussion

For the first time, we prospectively investigated whether a diagnostic high MI CDUS protocol can alter tumor perfusion to improve the therapy outcome of neoadjuvant chemotherapy in breast cancer. With our diagnostic high MI CDUS protocol, we did not detect changes in the vascular morphology and function of breast cancer in patients. However, the application of this protocol in murine TNBC resulted in a significantly reduced tumor rBV, reduced microbubble velocity, and a lower amount of perfused blood vessels. Furthermore, we found differences in tumor morphology between the two species, possibly explaining the divergent effects of high MI CDUS.

Clinically, diagnostic CEUS commonly uses nonlinear acoustic responses of microbubbles that already occur at acoustic pressures below 200 kPa 29. However, microbubbles can also be visualized during their destruction using higher acoustic pressures when their disintegration is detected as Doppler signals. Both methods are suitable for investigations of breast lesions in patients. In the present study, we used Power Doppler ultrasound (7 MHz, MI 0.8) in combination with SonoVue^®^ microbubbles to investigate the effects of destructive CDUS on breast cancer vascularization during neoadjuvant chemotherapy.

Multiple preclinical studies 2, 3, 30-35 and one clinical study 8 aimed to enhance the antitumor efficacy of therapeutic agents using ultrasound and microbubbles in peripheral solid tumors. However, the ultrasound parameters, treatment time, contrast agent, type of tumor, and experimental read-out vary substantially. Although most studies were performed at low frequencies of 1-2 MHz, a direct comparison is difficult due to the wide range of variables between the different groups (Supplementary Tables S4 & S5). Next to the frequency and pressure that are applied on the tumor, pulse length, duration and duty cycle also influence the behavior of microbubbles resulting in different biological effects on the tumor tissue. Thus, for a systematic discussion despite the highly variable settings, we roughly categorize the experiments in approaches using sonopermeation to enhance drug delivery in tumors without damaging the vasculature 2, 3, 8, 30, 31, 35 and strategies that focus on improving retention of drugs by inducing a vascular breakdown 32-34.

In experiments focusing on improved drug delivery by sonopermeation, growth inhibition of murine prostate and pancreatic tumors 30, 35, as well as a prolonged survival of patients with pancreatic tumors 8 could be shown upon employing microbubbles plus ultrasound already at a low MI (0.2-0.4), where stable oscillation of microbubbles occurs. However, none of these studies directly assessed drug accumulation in tumors. Nevertheless, for breast cancer xenografts in mice there are publications reporting an enhanced drug uptake and improved therapy outcome when destroying the microbubbles with an MI between 0.5 and 1.0 (at 1 MHz) 3, 31. Furthermore, an improved drug accumulation was also shown for an ultrasound protocol with a higher pressure and longer pulses (MI: 2, 1 MHz, 2 MPa, 5 s pulse length) in mouse neuroblastoma 2. Interestingly, while vascular damage was observed at an MI above 1 in the breast cancer xenografts 3, 31, no tissue damage was found in mouse neuroblastoma with a higher pressure of 2 MPa 2, indicating that the effects are tumor type-dependent.

The alternative approach to induce a vascular breakdown to trap chemotherapeutics was successfully demonstrated in murine breast 32, prostate 33, and liver 34 tumors. All of these studies were performed at high acoustic pressures of 1.65-3 MPa, where destruction of microbubbles is assumed. It could be shown that tumor perfusion was reduced 32-34 and that the overall amount of chemotherapy accumulation in tumors was enhanced 34.

In the present study, high MI CDUS was applied with a high MI of 0.8 and short pulses of 8 µs which, according to comparable parameters reported in the literature, does not induce severe tissue damage. Furthermore, we moved the transducer over the tumor and therefore reduced the time that the ultrasound can interact with the tissue at one location. We did not detect any effects of high MI CDUS on tumor perfusion by conventional time-intensity curve analysis. As a histological confirmation was not possible (45% of patients had pCR after completion of neoadjuvant chemotherapy), mULM was used to identify discrete changes in the vascular morphology and function. However, also this sensitive post-processing technique 22 did not depict morphological and functional changes in tumor vascularization. Partially, this might be explainable by using SonoVue^®^, which has a very broad size distribution, with 80% of the microbubbles ranging between 3 - 9 µm in diameter 29, 36. The resonance frequency of microbubbles strongly depends on their size, and the strongest oscillation occurs when a microbubble is excited close to its resonance frequency 29. In the case of SonoVue^®^, this leads to a wide range of frequencies (approx. 1-4 MHz) that can be used to induce nonlinear oscillation, which is favorable for clinical diagnostic applications at low acoustic pressure (below 200 kPa 29). However, this characteristic can be a limitation for the application in microbubble-assisted ultrasound treatment, as the acoustic behavior cannot be optimally controlled, and a prediction of bio-effects is difficult. Therefore, monodisperse microbubbles would enable better adjustment of ultrasound parameters and would consequently be favorable for sonopermeation.

Despite the lack of morphological and functional changes in the tumor vascularization, a slight but non-significant delay in tumor size reduction was observed in the high MI CDUS group during the first chemotherapy cycles. Although this may well be the result of undersampling, we decided to stop patient recruitment as it was clear that the intended positive effects on tumor perfusion were negligible. It is worth mentioning that we did not expect to observe significant improvements in the therapeutic outcome as the chemotherapy treatment regime, e.g., for TNBC with 4 cycles of epirubicin + cyclophosphamide every 2-3 weeks followed by 12 weekly cycles of paclitaxel combined with carboplatin is known to cause pCR in ~ 50% of patients 37. Thus, a very high number of patients would be required to observe significant improvements in therapy outcome. Rather, we intended to investigate whether high MI CDUS can cause significant perfusion changes during chemotherapy infusion or not. As discussed above, our mULM analysis results did not indicate any vascular effects.

The observation towards a somewhat slower response of high MI CDUS treated tumors to CTx has made us want to better understand the biophysical effects of our high MI CDUS protocol on tumor tissue and, therefore, we performed experiments on mice with TNBC using the same settings.

Interestingly, opposed to the results obtained in patients, post-processing with mULM revealed a significantly reduced rBV and microbubble velocity directly after high MI CDUS in mouse TNBC. Histological analyses confirmed a significant reduction in the number of perfused tumor blood vessels. Despite the reduced perfusion after high MI CDUS, the amount and distribution of carboplatin were comparable to untreated tumors. Possible explanations for this are that carboplatin accumulated in the tumor before the vascular breakdown occurred, as the carboplatin infusion was initiated shortly before applying high MI CDUS. Additionally, carboplatin was mostly detected close to larger and more mature vessels, which might be less affected by oscillating or collapsing microbubbles. This hypothesis is also supported by other studies in hepatocellular carcinoma 34 and the brain 38. Thus, the smaller disrupted vessels may have only contributed to a minor extent to the overall carboplatin accumulation, whereas drugs that accumulate in the considerably large extravascular space of larger vessels can still slowly diffuse through the stromal compartment towards necrotic areas and tumor cells might be the dominant delivery mechanism.

The question arises why we did not observe a vascular breakdown in human tumors. Although we used comparable ultrasound protocols to treat murine and human tumors, some differences between the experimental settings between mice and humans need to be mentioned as limitations of this study. First, murine tumors (and especially orthotopic breast tumor models) are located closer to the body surface, resulting in less tissue between tumor and transducer and thus higher energy deposition. The attenuation coefficient in breast fat tissue is 0.73 dB/cm/MHz 39, which results in an attenuation factor of 5,84 in 3 cm depth at 7 MHz. Additionally, the volume that was intravenously injected was higher in mice (human 0.01% of total blood volume versus mice 1.5% of total blood volume for each injection) which can alter the tumor perfusion by increasing the blood pressure. Another explanation for the conflicting findings is the different morphology of murine versus human TNBC. Murine 4T1 tumors grow very fast and do not develop excessive extravascular stroma, whereas human TNBC can be desmoplastic 40. This connective tissue potentially stabilizes blood vessels towards mechanical damage. Additionally, in mice, tumor blood vessels are approximately ten times smaller than those of human tumors. In smaller vessels, the effects of high MI CDUS on the endothelium are expected to be much stronger, as the collapse of microbubbles occurs closer to the vessel wall 34. This hypothesis may also explain the results of Dimcevski et al. on pancreatic carcinomas that, like our murine tumors, are known to have more and smaller vessels than human breast carcinomas 41. In patients with pancreatic carcinomas, contrast-enhanced ultrasound treatment during CTx infusion 8 improved therapy response compared to a historical patient cohort. These findings lead to the conclusion that close contact of the microbubbles to the endothelium might be necessary to influence the tumor perfusion. This could be achieved either by increasing the microbubble concentration within one injection, applying acoustic radiation forces to push the microbubbles towards the vessel wall, by using targeted microbubbles that bind to tumor endothelium, or by using microbubbles with a stiffer shell, as these are more likely to marginate to the vessel wall 42. Furthermore, ultrasound settings that provoke a stable and strong microbubble oscillation might be favorable to increase the vascularization and permeabilisation of tumor vessels. Stably oscillating microbubbles can interact with endothelial cells for a longer period of time compared to collapsing microbubbles which only once generate high energies via shock wave formation or microjets 43.

In summary, we here present a prospective clinical evaluation of high MI CDUS treatment for enhancing tumor vascularization to improve neoadjuvant chemotherapy in breast cancer. Regarding the various ultrasound settings that can be adjusted (e.g., frequency, pressure, duty cycle), we show that the current destructive ultrasound setting (7 MHz, MI 0.8, 18 min treatment time) is not suitable to improve tumor perfusion or response to neoadjuvant chemotherapy in patients, the latter even tending to be prolonged. We are aware that patient numbers are low but it is unlikely that a larger cohort of patients would have elucidated a clinically meaningful benefit of the ultrasound intervention. This is particularly true when taking our preclinical findings into account, where the high MI CDUS setting even leads to a partial vascular disruption. This stronger response of the preclinical than the clinical tumors may be explained by differences in their vascular and stromal composition. Only the combination of preclinical and clinical examinations in the present study enabled the identification of the tumors' vascular and stromal characteristics as an important factor for a successful application of microbubble assisted ultrasound treatment. Thus, there seem to be a small range in which high MI CDUS is either purely diagnostic, improves perfusion and vessel permeability, or induces vascular ablation. Intense research is still required to identify ideal ultrasound settings and microbubble formulations. In this regard, ultrasound protocols inducing a strong oscillation might be favorable to induce effects in highly desmoplastic tumors. Additionally, the effects may also strongly depend on the tumor tissue characteristics, which should be an integral part of future biophysical ultrasound examinations and enable the identification of suitable tumor entities and the preselection of responding patient subpopulations.

## Supplementary Material

Supplementary methods, figures and tables.

## Abbreviations

4T1: murine triple negative breast cancer cells
CDUS: contrast-enhanced Doppler ultrasound
CEUS: contrast-enhanced ultrasound
CTx: chemotherapy
FELASA: Federation for Laboratory Science Associations
H&E: Hematoxylin & Eosin
Her2: human epidermal growth factor receptor 2
ICP-MS: Inductively coupled plasma mass spectrometry
LANUV: German State Office for Nature, Environment and Consumer Protection
MCMCDA: Markov chain Monte Carlo data association algorithm
MI: mechanical index
mULM: motion-model ultrasound localization microscopy
NaCl: Sodium chloride
NCC: normalized cross-correlation
pCR: pathological complete response
PE: peak enhancement
rBV: relative blood volume
RPMI 1640: Roswell Park Memorial Institute 1640
RWTH: Rheinisch Westfälisch Technische Hochschule
TI: thermal index
TNBC: triple negative breast cancer
