# Supplementary material for: Effects of contrast-enhanced ultrasound treatment on neoadjuvant chemotherapy in breast cancer
Source: Theranostics. 2021 Sep 21;11(19):9557–70. doi: 10.7150/thno.64767 (PMC8490514; doi:10.7150/thno.64767)
Supplement: Supplementary file 1 — Supplementary methods, figures and tables. [file thnov11p9557s1.pdf]

Supplementary information:

Effects of contrast-enhanced ultrasound treatment on neoadjuvant chemotherapy in breast cancer

Anne Rix[1*], Marion Piepenbrock[2*], Barbara Flege[3], Saskia von Stillfried[4], Patrick Koczera[1,5], Tatjana Opacic[1], Nina Simons[2], Peter Boor[4], Sven Thoröe-Boveleth[6], Roel Deckers[7], Jan-Niklas May[1], Twan Lammers[1], Georg Schmitz[2+], Elmar Stickeler[3+], Fabian Kiessling[1+]

Chemotherapy treatment scheme

Patients diagnosed with Her2neu positive tumors received twelve weekly cycles with paclitaxel (80 mg/m^2 i.v.) combined with 4 times trastuzumab (8 mg/kg i.v.) + pertuzumab (840 mg/kg i.v.) every third cycle, followed by four cycles of epirubicin (90 mg/m^2) combined with cyclophosphamide (600 mg/m^2) every two to three weeks.

Patients with hormone receptor positive tumors received twelve weekly cycles of paclitaxel (80 mg/m^2 i.v.) followed by four cycles of epirubicin (90 mg/m^2) combined with cyclophosphamide (600 mg/m^2) every two to three weeks.

Histological and immunofluorescence analyses of human tumor samples

Paraffin-embedded punch biopsies of human TNBC were cut in 3 µm thick slices (HM430, ThermoFisher, Waltham, USA), dried overnight at 60 °C, and deparaffinated. Connective tissue was stained using the trichrome stain kit (ab150686, Abcam, Berlin, Germany). For fluorescent staining of blood vessels, antigen demasking was performed for 20 min at 98 °C in AR6 buffer (AkoyaBiosciences, Marlborough, USA). Blood vessels were stained using mouse anti-human CD31 antibody (4 µg/ml, SkyTec Laboratories, Logan, USA, #RA0259) followed by horse Anti-Mouse IgG Antibody (H+L), Peroxidase (5 µg/ml, Vector Laboratories, Burlingame, USA, # PI-2000) and TSA Cyanine 3 (1:50, PerkinElmer, Waltham, USA, #NEL704A001KT). Nuclei were counterstained with 4’6-diamidino-2-phenylindole (0.5 µg/ml DAPI, Merck, Darmstadt, Germany). Smooth muscle actin was stained using rabbit anti-human SMA antibody (0.25 µg/ml, Abcam, Berlin, Germany, #ab32575) followed by goat Anti-rabbit IgG Antibody (H+L), Peroxidase (0.25 µg/ml, Abcam, Berlin, Germany, #ab97080) and TSA Cyanin 5 (1:50, PerkinElmer, Waltham, USA, #NEL705A001KT).

After neoadjuvant chemotherapy, the tumor area was surgically resected, underwent a macroscopical evaluation, formalin-fixation (4%), and sampled for histological assessment. Samples were dehydrated and paraffin embedded. Tissue samples were cut in 3 µm thick slices and stained with Hematoxylin & Eosin. Whole slide pictures were acquired using an Aperio Slide Scanner (Leica, Wetzlar, Germany).

ICP-MS analysis of carboplatin contents in murine tumor samples

Murine tumors were collected and bisected. One half was embedded in Tissue Tek® O.C.T. Compound (Sakura, Alphen aan den Rijn, Netherlands) and stored at -80 °C. The tissue of the other tumor half was lysed using the radioimmunoprecipitation assay (RIPA) buffer (Merck, Darmstadt, Germany), and the platin concentration in the tumor tissue was assessed by inductively coupled plasma mass spectrometry (ICP-MS). To achieve this, 0.5 ml nitric acid (Suprapur, 65%, Merck, Darmstadt, Germany) was mixed with an equal amount of each lysate sample. After complete dissolution of the particles, the mixture was filled up to 5 ml with deionized water and analyzed using an ICP-MS machine (8900-QQQ, Agilent, Waldbronn, Germany). Rhodium (1 mg/L, Merck, Darmstadt, Germany) was used as an internal standard for error correction.

The tissue distribution of Pt^{195} was assessed on 10 µm thick tumor slices. First, the rhodamine-lectin signal was acquired using the Vectra® 3 imaging system (Perkin Elmer, Waltham, USA) on the whole slide to visualize perfused tumor vessels. Afterward, the Pt^{195} content was measured on the same tumor slice using the Agilent 8900 ICP-MS (Agilent Technologies, Waldbronn, Germany) equipped with a New Wave NWR213 Laser (Elemental Scientific, Omaha, USA).

Histological and immunofluorescence analysis of murine tumor samples

Frozen tumor samples were cryosectioned into 8 µm thick slices (CM3050S, Leica, Wetzlar, Germany). Three sections from different tumor locations were fixed with methanol (80%, Merck, Darmstadt, Germany) and acetone (Merck, Darmstadt, Germany). Blood vessels were stained with rat-anti mouse PECAM-1 monoclonal antibodies CD31 (50 µg/ml, BD Biosciences, San Jose, USA, #553370, RRID: AB_394816) followed by donkey-anti-rat IgG (H+L) (0.001 mg/ml, Dianova, Hamburg, Germany #712-546-153). Nuclei were counterstained with 4'6-diamidino-2-phenylindole (0.5 µg/ml DAPI, Merck, Darmstadt, Germany). Smooth muscle actin was stained using a biotinylated monoclonal alpha-SMA antibody (19 µg/ml, Progen, Heidelberg, Germany, # BK61501) followed by Streptavidin-AMCA (9 µg/ml, Dianova, Hamburg, Germany, # 016-150-084).

Five fluorescent images per tumor slice were captured using a fluorescent microscope (Axio Imager.M2, Zeiss, Göttingen, Germany) and quantified using the ImageJ2 software [Rueden BMC Bioinformatics 2017]. The percentage of area fraction with co-localized CD31 and rhodamine-labeled Ricinus Communis agglutinin I signal was determined to quantify perfused vessels. Also, for comparison of vessel sizes between mouse and human TNBC, histological specimens of 10 untreated 4T1 tumors grown in the same mouse strain were analyzed.

Supplementary table S1: Design of the clinical study.

**Study design**	monocentric, prospective, randomized-controlled, two-armed open observational study
**Inclusion criteria**	- histologically confirmed primary breast cancer, including all subtypes<br>- neoadjuvant chemotherapy<br>- of legal age<br>- written consent<br>- legally competent, mentally able to follow the instructions of the study staff
**Exclusion criteria**	- hypersensitivity/ allergy against sulfurhexafluorid, Macrogol 4000, distearoylphosphatidylcholine, dipalmitoylphosphatidylglycerol – sodium palmitic acid<br>- right-left shunt<br>- severe pulmonary hypertension (pulmoarterial pressure > 90 mmHg)<br>- uncontrolled systemic hypertension<br>- acute respiratory distress syndrome<br>- pregnancy<br>- persons who are accommodated in an institution on official or judicial instruction<br>- persons who are in a dependent or employment relationship with the sponsor or investigator<br>- alcohol or drug abuse<br>- expected non-compliance

**Primary outcome**	tumor size reduction when combining neoadjuvant chemotherapy and contrast-enhanced diagnostic ultrasound compared to neoadjuvant chemotherapy (reduction in %), pCR (histopathological complete remission) and cCR (clinical complete remission, measured using ultrasound)
**Secondary outcome**	- proliferation rate of tumor cells (Ki67 protein analysis)<br>- narrowing of the invasion front of the tumor<br>- intra-tumoral macrophage density<br>- tumor vascularization<br>- chemotherapy tolerance

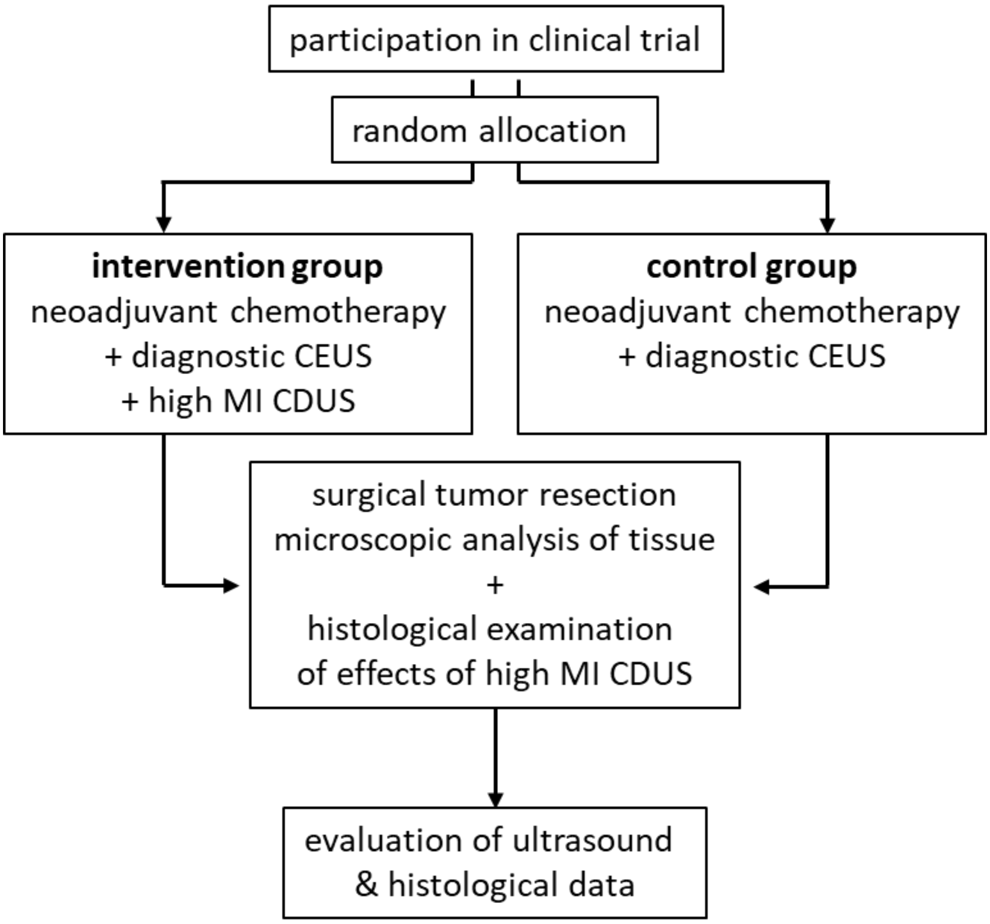

Supplementary figure S1: Clinical trial flow chart.

Supplementary table S2: Patient data before neoadjuvant chemotherapy.

patient	before chemotherapy							
	**tumor type***	**tumor volume (mm^3)**	**ER**	**PR**	**Her2**	**Ki67 (%)**	**subtype**	**grade**
**1**	TNBC	25611	0	0	FISH-	83	no special type	G3
**2**	Hormon$^+$	15504	12	12	0	30	invasive lobular	G2
**3**	Her2neu$^+$	117519	2	0	3+	60	No special type	G3
**4**	TNBC	21122	0	0	FISH-	80	no special type	G3
**5**	TNBC	13433	2	0	1+	20	no special type	G3
**6**	TNBC	3957	0	0	0	75	no special type	G2
**7**	TNBC	2090	0	0	0	76	no special type	G2
**8**	Her2neu$^+$	8620	0	0	3+	40	no special type	G2
**9**	Hormon$^+$	24001	4	0	0	80	no special type	G3
**10**	TNBC	21419	0	0	0	90	medullary carcinoma	G3
**11**	TNBC	7623	2	0	1+	95	no special type	G3

ER = estrogen receptor, PR = progesterone receptor, Her2 = Her2neu receptor

*Tumor type classification according to Remmele und Stegner (1987): Pathologe 8:138-140 [Recommendation for uniform definition of an immunoreactive score (IRS) for immunohistochemical estrogen receptor detection (ER-ICA) in breast cancer tissue]

Supplementary table S3: Patient data after neoadjuvant chemotherapy.

patient	after chemotherapy					
	**ER**	**PR**	**Her2**	**Ki67 (%)**	**regression grade**	**TNM**
**1**	not performed				4	ypT0, ypN0 (0/2), L0, V0, R0
**2**	not performed			20	0	ypT3, ypN1a (1/9), L0, V0, Pn0, R1
**3**	0	0	3+	40	1	YpT2(m), ypN1a(3/3), L1, V0, Pn0, R1; ypTis(DCIS), R1
**4**	not performed				4	ypT0 ypN0 (0/9) R0
**5**	0	0	0	50	1	ypT1b ypN0 (0/4 sn) L0 V0 R0. pTis R0.
**6**	not performed				4	ypT0, ypN0 (0/1 sn), L1, V0, R0.
**7**	0	0	0	80	1	ypT1b ypN0 (0/2sn) L0 V0 R0, ypTis R0
**8**	not performed				4	ypT0, ypN0(0/2), L0, V0, R0
**9**	2	0	0	not performed	2	ypT1a, pNx, L0, V0, R0, ypTis(DCIS HG), R0
**10**	1	0	0	90	1	ypT1c, ypN0(0/2 sn), L0, V0, R0.
**11**	not performed				4	ypT0, ypN0 (0/2 sn), L0, V0, R0.

ER = estrogen receptor, PR = progesterone receptor, Her2 = Her2neu receptor

Regression grade classified according to Geburtshilfe Frauenheilkd. 1994 Oct;54(10):552-8. [Histologic regression of breast cancer after primary (neoadjuvant) chemotherapy]. Sinn HP1, Schmid H, Junkermann H, Huober J, Leppien G, Kaufmann M, Bastert G, Otto HF.

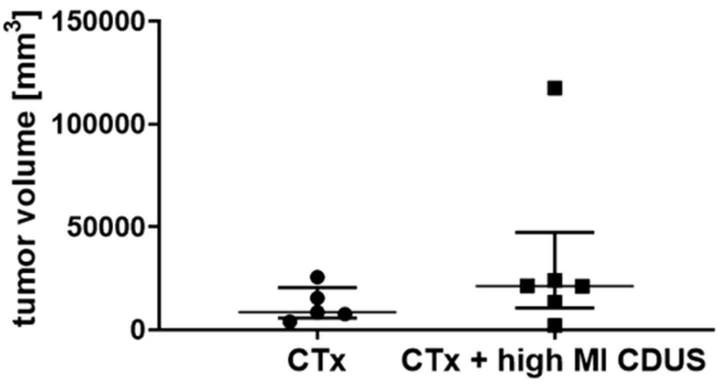

Supplementary figure S2: Initial tumor size in patients. The tumor sizes of CTx and CTx + high MI CDUS treated patients was comparable at the start of the clinical study. Data presented as median and interquartile range.

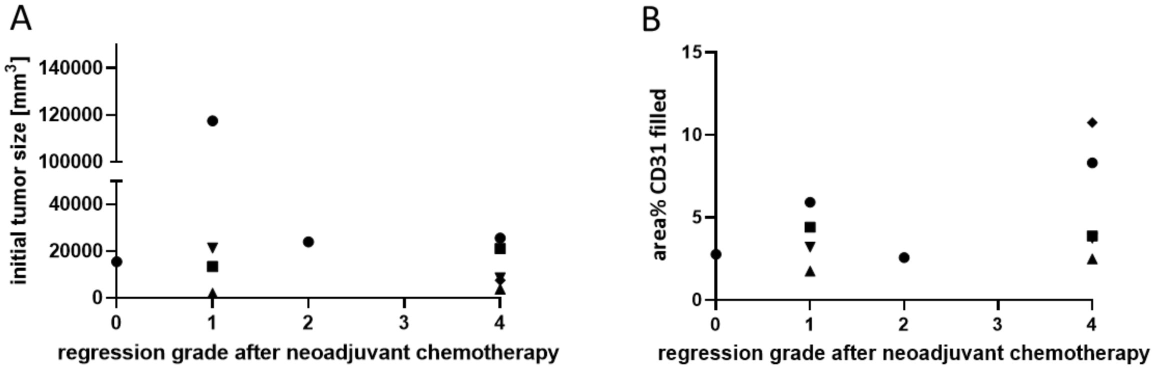

Supplementary figure S3: Relations between tumor characteristics and therapy response. The comparison of the A: initial tumor size and B: initial tumor vascularization with the regression grade after neoadjuvant chemotherapy did not reveal a relation of these parameters in our small patient cohort.

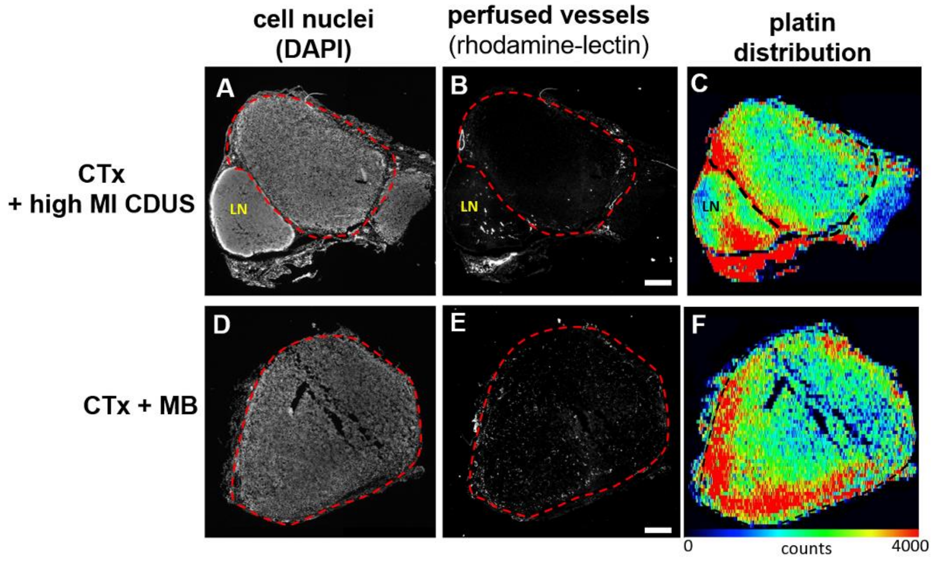

Supplementary figure S4: Pt^{195} distribution in murine TNBC. LA-ICP-MS measurements of PT^{195} revealed that carboplatin can be found in the whole tumor slice with the highest concentration located around areas containing bigger vessels in both, CTx + high MI CDUS (upper panel) and CTx + MB (lower panel) treated tumors. Surprisingly, although the number of perfused vessels was significantly reduced in CTx + high MI CDUS treated tumors, carboplatin can be found in the avascular tumor center. Possible reasons could be that carboplatin accumulated in the tumor already before the vascular breakdown as the carboplatin infusion was initiated shortly before the application of high MI CDUS. Furthermore, larger vessels are less affected by high MI CDUS and these vessels have a considerably large extravascular space in which the carboplatin can still accumulate and then, slowly diffuse into the tumor tissue A + D: Cell nuclei are stained with DAPI. B + E: Perfused vessels are stained by i.v. injection of rhodamine-labelled Lectin. C + F: Carboplatin distribution measured by LA-ICP-MS. Scale bar = 100 µm, LN = lymph node.

Supplementary table S4: Detailed ultrasound settings of all studies aiming to enhance drug delivery without damaging the tumor vasculature

study	frequency (MHz)	acoustic pressure (MPa)	mechanical index	pulse	duty cycle (%)	treatment time	additional information	contrast agent
Bellary 2020[2]	1	2		4 cycles: 5 s on/ 5 s off (5x); then 30 s gap		~5,5 min	acoustic intensity 3 W/cm^2	phospholipid microbubbles 1x10^9 microbubbles
Snipstad 2017[3]	1	0.1 - 1		10000 cycles: 10 ms every 100 ms	2.5	2 min	pulse repetition frequency 0.5 Hz	cyanoacrylate microbubbles self-assembled from nanoparticles 2.6 ± 1.3 mm 10 mg/ml nanoparticles, 200 µl
Dimcevski 2016[8]	1.9	0.27	0.2	4 cycles (2.1 µs) every 21 ms	0.003	31,5 min	acoustic intensity 0.25 mW/cm^2	SonoVue® 9 x 0.5 ml
Kotopoulis 2014[30]	1	0.2	0.2	40 µs every 100µs	40	10 min	acoustic intensity 688 mW/cm^2	SonoVue® 50 µl
Sorace 2012[31]	1		0.1, 0.5, 1, 2		20	5 min	pulse repetition frequency 5 s (0.2 Hz)	Definity® 30 µl
Bressand 2019[35]	1	0.4			40	3 min	pulse repetition frequency 10 kHz (100 µs intervals)	Bracco BG8214/ BG8610 70 µl
Rix 2021	7	2.1	0.8	16 pulses (0.008 ms each) spaced 0.08 ms apart each 0.5 ms (estimated)	7 (estimated)	18 min		SonoVue® 6 x 0.5 ml

Supplementary table S5: Detailed ultrasound settings of all studies aiming to improve drug retention by inducing a vascular breakdown

study	frequency (MHz)	acoustic pressure (MPa)	mechanical index	pulse	duty cycle (%)	treatment time	additional information	contrast agent
Todorova 2013[32]	1	1.65		50 x 0.1 ms pulses spaced 1ms apart each 20 s		3x 3 min		Definity® 60 µl/kg
Goertz 2012[33]	1	1.65		50 x 0.1 ms pulses spaced 1ms apart each 20 s		3x 3 min		shell: sorbitan monostearate (Span 60) and polysorbate 80 (Tween 80); gas: octofluropropane 3.8 ± 2.13 µm; 2.1 x 10^5 bubbles/g BW
Keller 2020[34]	1.6	2-3		4 cycles: 5 s on/ 5 s off (6 x); then 90 s gap		6 min	pulse repetition frequency 50 Hz	SonoVue® 50 µl
